# Sleep Health Issues for Children with FASD: Clinical Considerations

**DOI:** 10.1155/2010/639048

**Published:** 2010-07-14

**Authors:** James E. Jan, Kwadwo O. Asante, Julianne L. Conry, Diane K. Fast, Martin C. O. Bax, Osman S. Ipsiroglu, Elizabeth Bredberg, Christine A. Loock, Michael B. Wasdell

**Affiliations:** ^1^Pediatric Neurology and Developmental Pediatrics, Child and Family Research Institute and BC Children's Hospital, University of British Columbia, 4500 Oak Street, Vancouver, BC, Canada V6H 3N1; ^2^Pediatrics, Asante Centre for Fetal Alcohol Syndrome, University of British Columbia, Maple Ridge, BC, Canada V2X 3C1; ^3^Department of Educational and Counselling Psychology and Special Education, University of British Columbia, Vancouver, BC, Canada V6T 2Z4; ^4^Associate Pediatrics, University of British Columbia and BC Children's Hospital, Vancouver, BC, Canada V6H 3N1; ^5^Child Health, Chelsea and Westminster Campus, Imperial College, SW7 2AZ London, UK; ^6^Developmental Pediatrics, University of British Columbia and Sunny Hill Health Centre for Children, Vancouver, BC, Canada V5M 3E8; ^7^BC Children's Hospital, Vancouver, BC, Canada V6H 3V4; ^8^Research Administration and Development, Fraser Health Authority, Surrey, BC, Canada V3R 7P8

## Abstract

This article describes the combined clinical experience of a multidisciplinary group of professionals on the sleep disturbances of children with fetal alcohol spectrum disorders (FASD) focusing on sleep hygiene interventions. Such practical and comprehensive information is not available in the literature. Severe, persistent sleep difficulties are frequently associated with this condition but few health professionals are familiar with both FASD and sleep disorders. The sleep promotion techniques used for typical children are less suitable for children with FASD who need individually designed interventions. The types, causes, and adverse effects of sleep disorders, the modification of environment, scheduling and preparation for sleep, and sleep health for their caregivers are discussed. It is our hope that parents and also researchers, who are interested in the sleep disorders of children with FASD, will benefit from this presentation and that this discussion will stimulate much needed evidence-based research.

## 1. Introduction

 Many children diagnosed with fetal alcohol spectrum disorders (FASD) have long-standing sleep disturbances which interfere with their daily activities, cognition, behaviour, health, and management. Without appropriate treatment of sleep difficulties, the effectiveness of all interventions may be markedly reduced. Yet, sleep health is so neglected that there is an absence of comprehensive literature on it and its management. The purpose of this article is to summarize the clinical experience of several professionals familiar with FASD and sleep disturbances in order to provide information to the caregivers and promote badly needed evidence-based research.

There is an increasing awareness of FASD among professionals, and FASD is now recognized to be a major health concern worldwide. It is a spectrum of effects caused by exposure to alcohol during gestation which effects include physical, neurological, behavioural, cognitive, and other disabilities, with lifelong implications. Alcohol, a powerful teratogen, can interfere with brain development throughout gestation resulting in a vast array of neurodevelopmental problems. The incidence of FASD is approximately 9.6/1000 live births in the North American population. In comparison, full fetal alcohol syndrome which requires characteristic craniofacial dysmorphology, growth restriction, and cognitive abnormalities, is only 1–3/1000 live births [1–7]. Many children with FASD have the same severity and complexity of cognitive deficits but without the characteristic physical features [8]. These children are often undiagnosed and their cognitive deficits tend to be misunderstood. Nowadays, many pregnant women who use alcohol smoke cigarettes and take cocaine, marijuana, and other drugs. Concurrent use of these agents, depending on their number and effects, increasingly interfere with brain development, especially with gray matter formation [9, 10]. 

Parental reports suggest that children with FASD frequently have persistent sleep problems but the exact prevalence is unclear. We are only aware of a single sleep survey that examined children with FASD for sleep onset delay, sleep duration, and other sleep disturbances [11]. One hundred caregivers of children aged 5–8 years participated in this study. Mean sleep duration was 7.4 hours and the mean sleep onset delay was 59 minutes. In the 7-day diary, 82 of the 100 caregivers reported other sleep problems such as night terrors (22), sleep walking (3), waking more than twice during the night (55), and day time fatigue (10). Thus, the findings agreed with the impressions of the caregivers that children with FASD frequently have sleep difficulties.

 The evolution of sleep disturbances in children with FASD has not been studied likely because of difficulties with the early diagnosis of this disorder as the characteristic dysmorphic features may not be fully apparent in early childhood [12]. Maternal self-report of alcohol consumption is often unreliable and some physicians are reluctant to diagnose FASD [13]. Also, the sleep disturbance of a young child only becomes a problem for the caregivers when their own sleep is severely disrupted and parents rarely ask for medical help until they have tried numerous home remedies. A retrospective review of hospital records of 50 neonates with FASD indicated symptoms of neonatal drug withdrawal in 30 infants which included disturbed sleep [14], but there was no follow-up. However, caregivers and clinicians familiar with FASD agree that sleep disorders tend to begin in the neonatal period. Systematic outcome studies of sleep disturbances have not been done either, but in our experience without treatment the sleep disorders tend to continue into adulthood. In this respect, children who were born with other severe neurological problems and experience sleep disturbances also appear to have an early onset of sleep difficulties which then become chronic, often lasting for years or a life-time. 

## 2. Sleep Disorders of Children with FASD

 The accurate diagnosis of a sleep disturbance is critical for appropriate management but a detailed discussion of this topic is beyond the scope of this article. There are publications on the sleep disorders of children without [15–18] and with neurodevelopmental disabilities [19]. The causes of sleep difficulties in children with FASD are frequently multifactorial because in addition to brain maldevelopment, sleep disturbances may be secondary to health problems, inadequate sleep hygiene, emotional and social issues. For example, the continued consumption of alcohol by the mother can lead to disturbed sleep of the infant because her breast milk contains alcohol [20]. While it may be difficult to immediately identify the causes of sleep difficulties in young children, these should become clear with follow-up. Understanding the home environment is important because sleep is strongly influenced by health, cultural, social, and economic factors and other considerations of the family; therefore a home visit is useful for more accurate diagnosis and also for better management. Unfortunately, little or no attention has been paid to the best management techniques of sleep disorders in children with FASD.

 During the last 20 years, numerous studies described the high prevalence rates of sleep disturbances affecting children with various forms of neurodevelopmental disabilities which publications were summarized in review articles [21, 22]. It became apparent that the sleep difficulties were related to the severity of cognitive loss and brain disturbance rather than to the specific diagnosis of disabilities [23]. For this reason, we consider the sleep disturbances of children with FASD to be similar to the sleep difficulties of children with other forms of severe cognitive loss and bilateral brain damage. Most of these sleep disturbances are described by the caregivers as difficulties falling asleep, frequent awakenings during the night for minutes or even hours, and early morning awakenings. These sleep difficulties fall under the diagnostic category of circadian rhythm sleep disorders which are defined as dissociations between the sleep-wake behaviours and the environment.

 The circadian rhythms, including sleep and pineal melatonin production, are modulated by the suprachiasmatic nuclei of the hypothalamus. These nuclei receive input in the form of light/darkness and also cognitive environmental information from the cerebral cortex which then influences the timing, duration, and quality of sleep. When perceptual and cognitive functions are significantly disturbed, dissociation may occur between the sleep-wake behaviors and the environment. Totally blind but intellectually normal individuals often experience free-running sleep/wake rhythms which usually manifest as a tendency to fall asleep and waking up later every day. These individuals produce normal amounts of melatonin but with a daily delay. In contrast, studies clearly show that children with severe cognitive loss due to any cause frequently have delayed sleep onset, multiple prolonged arousals during the night, and early morning awakenings and these are the results of abnormal pineal melatonin production/secretion. Indeed, melatonin therapy given at bedtime may fully correct the circadian rhythm sleep disorders in these children [24]. Since the cerebral cortex has a modulating influence on the sleep/wake promoting centers of the hypothalamus, any excitatory activity, vigorous physical exercise, or anxiety before bedtime can delay pineal melatonin secretion and with it sleep onset, while relaxing activities do the opposite. In some children with severe neurodevelopmental disabilities, the excitatory activities may disturb the sleep/wake promoting centers of the hypothalamus and reduce melatonin production/secretion during the entire night, giving rise not only to difficulties falling asleep but also to frequent nocturnal arousals and early morning awakenings [23].

## 3. Neurocognitive and Behavioural Manifestations of Impaired Sleep

 It is well accepted that inadequate sleep presents not only as daytime sleepiness but as behavioural and neurocognitive dysregulation and impaired health. The behavioural manifestations include hyperactivity, aggressiveness, inattentiveness, impulsivity, depression, and other mood disorders [7, 25]. In cognitive functioning, deficits are seen in verbal fluency, comprehension, in abstract and deductive reasoning, planning, flexibility, inhibition, problem solving, attentiveness, vigilance, memory formation and motor skills, in addition to numerous other difficulties [26, 27]. Insufficient sleep may simultaneously affect multiple neurological and cognitive activities. Long-lasting sleep loss during critical developmental periods is especially harmful because it deprives young children of environmental exposure required for healthy cognitive and motor development and their ultimate developmental potentials may not be reached [28]. Researchers are beginning to understand the cellular and metabolic changes resulting from chronic sleep loss which can even lead to neuronal loss and permanent cognitive deficits [29, 30]. When children sleep poorly, the sleep of their caregivers and of other children are also affected [31]. Persistent sleep difficulties in all age groups are associated with an increased prevalence of diabetes [32], obesity [33], cardiovascular problems [34], depression and suicide attempts, more so in adolescents [35]. 

 Many of the deficits seen from sleep loss are also observed in children with FASD who do not have sleep difficulties [2–5]. Parents and educators agree that children with FASD, with or without sleep difficulties, may adapt differently cognitively and behaviourally to the environment, than typical children. Their neuropsychological profiles and behaviours are highly variable [4]. They may have difficulties with attention, memory, cognitive flexibility, generalization from one experience to another, low frustration tolerance, and exhibit unexpected emotional reactions in various situations [36]. It is often puzzling to caregivers why they respond and behave in unusual ways in one environment when in another they may be friendly, charming, kind, cooperative, and talkative. They may be repeating certain words and phrases or specific behaviours and exhibit obsessive-compulsive traits. In later age, they often have difficulty with the legal system as well [37–39]. 

Caregivers come to understand that children with FASD are not bad but that their cognition has been effected by prenatal alcohol exposure and as a result they may respond to their environment in different and unpredictable ways. It is well accepted by parents that in order to promote better behaviours of their children with FASD, modifying the environment is critical and that routines and protection from overstimulation at home, in school, and in social situations are most important. 

## 4. Sleep Promotion Activities

 According to the medical literature, the first step in the treatment of sleep disorders is the introduction of sleep hygiene [17, 19] which is defined as promotion of optimal sleep health practices through environmental management. It includes scheduling of sleep, improved sleep environment, and the use of various sleep-promoting practices. It needs to be strongly emphasised that sleep hygiene practices should be individually tailored for children with neurodevelopmental disabilities according to their cognitive deficiencies and health difficulties and may be challenging to implement [28]. However, based on our clinical experience, sleep hygiene interventions frequently fail to correct the sleep disturbances of children with severe neurodevelopmental disabilities partly because of their impaired understanding of environmental cues [24, 40]. Currently, there is no documented research on the promotion of sleep health for children with FASD and it is unclear how frequently sleep hygiene interventions are effective. 

 Sleep promotion activities and cues are numerous, complex, and interrelated. Sleep health practices are grouped under Sleep Environment, and sleep Scheduling and Bedtime Activities but the sleep needs of the caregivers and of the whole family must not be ignored either. The following discussions will explore the principles of sleep hygiene interventions for children with FASD in respect to the above categories.

## 5. Sleep Environment

Due to the widespread brain injury patterns in children with FASD, the capacity of the perceptual neural mechanisms appears to be limited. This may explain why exposure, even to minor stimuli at night, could be stressful and may lead to rapid sensory overload. Based on parental observations, there are many such examples [36, 41]. Children with FASD are commonly oversensitive to sudden or persistent noises, to loud music or voices, and to certain sounds which may not even be noticed by typical children. The behavioural responses could manifest in temper tantrums, unusual behaviours, or simply in an inability to cope. At night earplugs or machines that produce white noise, and thereby minimize disturbing sounds from the environment, may be helpful. Like individuals with cortical visual impairment [42], children with FASD can be light-sensitive, leading to excessive tearing and headaches. Fluorescent lights and glare are especially bothersome. Therefore, the lights at home may need to be adjusted. In the bedroom, pictures or complex patterns on walls, furniture, and linens may also result in sensory overload. This phenomenon is referred to as a “crowding effect” in vision research [43]. The same problem is evident when children with FASD are surrounded by a large number of people. Parents often observe that it is more calming for children when the bedroom has minimal furniture and the walls and the bedding have a uniform, nonexciting colour. It is also better if most toys are removed from the bedroom and clutter' is avoided.

The same type of oversensitivity is seen to tactile stimuli. According to parents, small tags on their pyjamas, elastics, and the weight of their blankets, wrinkles, or different textures in the bedding may annoy them. They may not be able to find a comfortable position for sleep. Interestingly, self-touch does not result in uncomfortable feelings. Tactile oversensitivity is seen in other neurological disorders as well [44]. In some individuals with FASD, olfaction may be disturbed [45], and perfumes, odours of soap, food, or cigarette may be unusually irritating to them. Certain tastes and textures may be bothersome and they may not be able to swallow their medications or lumpy food and easily gag or vomit. Their responses to bedroom temperatures may also be different. It is not surprising that the parents have to be observant in order to understand their behaviours.

According to parents, most children with FASD have difficulty deviating from their acquired skills. They are rigid and resistant to change and even slight rearrangement of the bedroom furniture may disturb them and cause sleep difficulties. Bedrooms should not be used for punishment or play and sharing their beds with the parents should be avoided when possible. Sleep environment promotes sleep when it is the same over time, secure, familiar, comfortable, and unexciting and when the children are proud to own their little place. A fear of the dark is not uncommon, because children with FASD often suffer from anxieties. A dim night light, which does not shine into the eyes of the child, can be useful, in spite of the commonly-given advice that any ambient light disturbs sleep. All of these factors need to be analyzed when designing an appropriate and individualized sleep environment for children with FASD.

## 6. Preparation for Sleep: Tips for Caregivers

For parents, preparing the child with FASD for sleep might be a difficult and time-consuming task. It is clear that calming activities promote sleep onset, while any form of excitation leads to a delay. The problem is that activities that relax typical children may be exciting to children with FASD; therefore, parents often have to learn by trial and error which behaviours are calming or exciting. Instead of getting advice from a friend or a relative with typical children, parents benefit most by talking to caregivers who are familiar with FASD. 

There is full parental agreement that the sequence of bedtime activities needs to be supervised and firmly enforced because children with FASD tend to have a poor concept of time and also have difficulty with order. It is important to note that they usually talk well, in spite of their impaired verbal perception, giving the impression that they fully understand what they are told, but this is often not the case. Children with FASD frequently experience failure and as a result try to cover up their deficiencies. They often appear defiant and stubborn because they misinterpret what they are told. Therefore, the parents should communicate with a soft voice, using short and simple sentences and frequent repetitions. When this approach fails, it is the experience of some caregivers that a series of picture cards posted on a wall could prompt the children to do certain tasks in sequence using the advantage of the generally better visual skills. For example, the picture cards may show a child, with the appropriate gender and age, having a bath, changing into pyjamas, brushing teeth, washing hands and face, listening to a story, praying, hugging, receiving a kiss, saying good night to a favorite toy nearby, and turning off the lights, and so forth. A good bedtime wind-down ritual can teach children with FASD to associate the routine with sleep onset. This type of strict routine is beneficial for improving sleep and also teaching them appropriate general hygiene, which is frequently poor in later life. 

The need for tailoring bedtime activities is best illustrated by storytelling. Stories must be carefully modified, not only according to the developmental age and to the degree of attentiveness, but also to specific cognitive abilities. The best principle to follow is “less may be better than more”. Therefore, the story needs to be simple, with short sentences, familiar words and repetitions. Because the integration of sensory information is impaired, books with sounds, textures, and smells may be more stimulating than calming and are best avoided.

Excessive physical and mental activities, beverages containing caffeine or chocolate, bright lights, excessive TV watching, video games, and even excited play with siblings before bedtime may cause a delay in sleep onset [28]. For typical children, the sleep hygiene literature emphasizes only appropriate presleep activities but for children with FASD, stress or excitement may need to be avoided during the entire day which may not be possible when they are in school. For typical children, exposure to a rich learning environment is suggested but for children with FASD this often leads to overstimulation and to disturbed sleep. Again, less stimulation is usually better than more. 

 Caregivers agree that children with FASD frequently do not know how to deal with their own emotions, anxieties, and lack of self-esteem. It is important for the parents to use appropriate relaxing techniques when their children show signs of overstimulation especially before bedtime. These techniques may consist of a warm bath or a shower, rocking, massaging, quiet music, singing, taking several deep breaths, and having a quiet time in a calm area without the feeling that it is punishment. During these times praise may be much more effective than scolding but it must be given immediately after a desired behaviour. Of course, preventing adverse behaviours should always be a priority. 

 Children with FASD often have medical problems such as allergies, esophageal reflux, sleep apnea, headaches, or painful conditions, which may affect their sleep. Medications prescribed for Attention Deficit Hyperactivity Disorder and epilepsy often delay the onset of sleep. All medications must be carefully monitored because they may have unexpected side effects on their sleep.

## 7. Sleep Scheduling

As pointed out earlier, a major feature of FASD is the marked variability of behaviours, which is common in all types of brain damage. Thus, one day their sleep is better and the next day it is worse. Sleep scheduling is not easy when children have variable sleep difficulties which from time to time make them so exhausted that they fall asleep before their regular bedtimes. Many children with FASD exhibit symptoms of Attention Deficit Hyperactivity Disorder [3] and they often have an impaired sense of time and poor organisational skills. Caregivers agree that rules, structuring, routine, and consistency are critical, not only for bedtime activities but for the entire day. The same time for going to bed and getting up in the morning must be consistently and carefully enforced, with minimal deviations, even during vacations and weekends. Similarly daytime naps require regulation on an individual basis.

## 8. Sleep Hygiene for the Caregivers

Children with FASD are overrepresented in foster placements, in care of biological relatives, or in adoptive homes [36, 41]. It is generally agreed that raising a child with FASD is usually a challenging undertaking. This requires constant work, diligent supervision and guidance, frequently leading to excessive caregiver stress, fatigue, sleep loss, burn-out, and depression. Birth, adoptive, or foster parents may have limited time for each other and for other family members for socialization. Yet, parenting responsibilities carry the high expectation of being kind and well prepared, understanding, patient, and consistent all the time. This is an enormously difficult task for the caregivers of children with FASD [46]. 

There are numerous parent resources but comprehensive instructions on sleep health management do not exist. Parents often state that professional guidance on sleep hygiene is lacking and the available lists of suggestions suitable for typical children are rarely helpful. As an example, respite care, when not offered in the home environment, may be most disruptive. The caregivers are placed in a position where they constantly have to educate others about the nature of FASD and their children's needs. When faced with the developmental concerns for their child, behavioural issues, repeated calls from school teachers, disagreements and their inability to offer optimal care to the whole family, parents may, indeed, perceive themselves to be “bad parents”. It is not surprising that they feel lonely, insecure, and bewildered [36, 41]. With an emphasis on the child, the parents' sleep needs are often ignored but parental sleep deprivation invariably results in compromised care for the children. Therefore, the promotion of sleep health must always include the caregivers. 

The families of children with FASD with or without difficult sleep problems often face health, economic, and social difficulties and, occasionally, serious addiction issues. Mothers may have to quit their jobs in order to cope with the 24-hour child care at home. Under these circumstances, it may not be realistic to expect that caregivers can carefully plan and carry out complex, long-term, and labour-intensive sleep hygiene programs for their child with FASD. What are the chances that the best sleep hygiene techniques will benefit the sleep difficulties of children when the parents are exhausted, the home is chaotic, and everyone is stressed? 

It is the collective experience of the authors that sleep hygiene interventions are increasingly harder to enforce and less effective in children with more severe cognitive loss and brain damage. For this reason, soon after patient contact, we often recommend bedtime melatonin supplementation to children with severe neurodevelopmental disabilities and circadian sleep disorders. Melatonin therapy is simple, highly effective, lacks significant adverse effects, and it acts quickly. Tolerance does not develop from continuous melatonin use and it is not addictive. Once melatonin therapy improves the child's sleep, it diminishes the burden for the entire family and while sleep hygiene practices are still necessary, most importantly, they are easier to achieve [24]. Therefore, when severe circadian rhythm sleep disorders are diagnosed, in many cases it may be the best practice to offer melatonin therapy and sleep promotion techniques simultaneously. A summary of sleep health practices that can be useful to clinicians and caregivers is presented in Table 1.

## 9. Future Research

This article was written because such information for parents and professionals does not exist. The purpose was to provide an introduction to the complexity of sleep difficulties and their management in FASD while focusing on sleep hygiene techniques. It is our hope that in the future, researchers who are interested in the causes and management of sleep disorders in children with FASD will benefit from this clinical presentation and that this discussion will stimulate much needed evidence-based research. The evolution of sleep difficulties and associated early behavioural features, the social interactions in the homes of young children with FASD, whether or not earlier diagnosis and treatment can prevent the development of sleep disturbances and many other areas discussed need to be carefully studied.

## Figures and Tables

**Table 1:** Recommendations for Sleep Health.

*General considerations*
(i) Children with FASD frequently have a melatonin deficiency which leads to disturbed sleep patterns
(ii) Sleep disturbances should be treated early and appropriately as they lead to neurocognitive behavioral and health difficulties
(iii) Intervention services may be ineffective when sleep deprivation is present
(iv) The functioning of children with FASD is highly variable; therefore developmental evaluations are helpful to understand their strengths and weaknesses
(v) Sleep hygiene practices designed for typical children are often not useful for those with FASD as interventions need to be tailored to individual abilities
(vi) Caregivers and involved professionals should work together in a team
(vii) Modifying the environment, protection from over-stimulation at home, in school and in social situations are important principles in the general management of children with FASD
(viii) The rich learning experience that is required for typical children may lead to over-loading and disturbed sleep for children with FASD
(ix) Sleep hygiene interventions are increasingly hard to enforce and less effective in children with more severe cognitive loss.

*Sleep environment*

(i) The children's reactions to the environment should always be carefully observed
(ii) The bedroom needs to be quiet, comfortable (temperature, non-irritating clothing and bedding), familiar, secure, consistent and unexciting (minimal furniture without clutter, strong odors, bright lights and colors)
(iii) Do not use the bedroom for punishment or play.

*Preparation for sleep*

(i) Calming behaviours and wind-down rituals promote sleep
(ii) Beverages containing caffeine or chocolate, excessive mental and physical behaviors, TV and video games should be avoided in the evening to minimize alertness and delayed sleep onset
(iii) Bedtime activities require supervision with emphasis on general hygiene which is often poor in later life.

*Sleep scheduling*

(i) Enforcing rules, structure, routine and consistency are important not just at bedtime but all day
(ii) Times for bed and getting-up need to be consistent, even during weekends and holidays
(iii) Melatonin replacement therapy for the child combined with sleep health promotion techniques may be useful to establish sleep scheduling.

*Sleep hygiene for the caregivers*

(i) Raising a child with FASD is a difficult task, thus the sleep health and the emotional needs of the caregivers must always be considered
(ii) Caregiver sleep patterns are linked to those of the child. Treatment of the child's sleep disturbance with melatonin may lead to better sleep health of the caregivers and reduced burden of care.
